# Sensitivity and Tolerance of Riparian Arthropod Communities to Altered Water Resources along a Drying River

**DOI:** 10.1371/journal.pone.0109276

**Published:** 2014-10-08

**Authors:** Kevin E. McCluney, John L. Sabo

**Affiliations:** School of Life Sciences, Arizona State University, Tempe, AZ, United States of America; University of Veterinary Medicine Hanover, Germany

## Abstract

**Background:**

Rivers around the world are drying with increasing frequency, but little is known about effects on terrestrial animal communities. Previous research along the San Pedro River in southeastern AZ, USA, suggests that changes in the availability of water resources associated with river drying lead to changes in predator abundance, community composition, diversity, and abundance of particular taxa of arthropods, but these observations have not yet been tested manipulatively.

**Methods and Results:**

In this study, we constructed artificial pools in the stream bed adjacent to a drying section of the San Pedro River and maintained them as the river dried. We compared pitfall trapped arthropods near artificial pools to adjacent control sites where surface waters temporarily dried. Assemblage composition changed differentially at multiple taxonomic levels, resulting in different assemblages at pools than at control sites, with multiple taxa and richness of carabid beetle genera increasing at pools but not at controls that dried. On the other hand, predator biomass, particularly wolf spiders, and diversity of orders and families were consistently higher at control sites that dried. These results suggest an important role for colonization dynamics of pools, as well as the ability of certain taxa, particularly burrowing wolf spiders, to withstand periods of temporary drying.

**Conclusions:**

Overall, we found some agreement between this manipulative study of water resources and a previous analysis of river drying that showed shifts in composition, changes in diversity, and declines in abundance of certain taxa (e.g. carabid beetles). However, colonization dynamics of pools, as well as compensatory strategies of predatory wolf spiders seem to have led to patterns that do not match previous research, with control sites maintaining high diversity, despite drying. Tolerance of river drying by some species may allow persistence of substantial diversity in the face of short-term drying. The long-term effects of drying remain to be investigated.

## Introduction

Human activities are dramatically altering the distribution of freshwater across the Earth’s surface and these changes may have important effects on both aquatic and terrestrial ecosystems worldwide [1]–[4]. Along the unregulated San Pedro River, in AZ, USA, groundwater and river waters have declined in recent decades, converting some once perennial reaches to reaches with only intermittent flows [5], [6]. Whereas the impacts of these water declines on aquatic animals [7]–[12] and on riparian (streamside) vegetation have been relatively well investigated [5], [13], [14], our understanding of the impacts on terrestrial animal communities remains limited.

Evidence of the effects of river drying on aquatic ecosystems could provide us with several hypotheses about how riparian animals might respond to river drying. For instance, stream and river drying events have been shown to have strong, long-term effects on aquatic community structure and diversity [7], [15], [16]. Additionally, even short-term drying events seem to reduce aquatic food chain length in rivers across the US [8]. However, refugia in the hyporheic zone and migration may modulate these effects, providing some degree of resilience, with community dynamics related to life-history strategies, e.g. [7], [11], [12], [17].

Rivers provide many important resources to terrestrial consumers. Many consumers rely on subsidies of emergent aquatic insects for energy and nutrients [18]–[20]. Similarly, some riparian herbivores rely on algal subsidies [21]. Additionally, the river water itself may be an important resource that could limit survival and performance [22]–[24]. This is especially true in arid lands and may also hold during droughts in mesic biomes when rivers are the sole source of free water [25], [26].

Here we focus on the effects of river drying on terrestrial riparian arthropod communities. Terrestrial arthropods play key functional roles in ecosystems, influencing rates of decomposition [27] and altering emergence of certain groups of aquatic insects in ways that depend on the identity of the consumer involved [28]. Arthropods are also near the base of the food web and thus are important for the support of higher consumers, including bird species found along the San Pedro River that attract tourism to the region [29].

Studies on other rivers in arid [30]–[32] and mesic [33]–[35] regions have examined the influence of flood alteration or flow regulation on terrestrial arthropods. Additionally, some research has investigated how desiccation tolerance influences species distributions along gradients of soil moisture and other habitat conditions [25], [26]. These studies found that carabid beetles are often particularly sensitive to alterations of flood regime, showing increases in abundance and diversity with flood events [31], [32] or shifts in community composition between rivers that differ in flood regime [35] or habitats that differ in flood disturbance [33]. Because many riparian carabids derive most of their nutrients from emergent aquatic insects, river drying may impact this group most severely by altering both food and water resources [19]. Some studies also suggest that spiders are often less influenced by changes to flow regime than many other groups of riparian arthropods [31], [36], despite the opposite reported in other studies [33] and the finding that riparian spider distributions often match differences in desiccation tolerance [25], [26]. The influence of river drying events specifically on riparian arthropods remains poorly studied, but see [37], [38].

Previous observational analyses along the San Pedro River, which experiences seasonal drying, found differences in predator abundance, familial community composition, familial diversity, and abundance of some groups of arthropods between dry and flowing river reaches [39]. Similar, but stronger patterns were observed for genera within the family Carabidae. Analyses of association of arthropods with environmental parameters in that study suggested that water resources were of prime importance in structuring the community. Although this previous work shed light on how river drying may influence arthropod communities, only a manipulative approach can provide direct causal evidence of the effects of water resources on arthropods. Thus, in this paper, we ask if previously observed differences between dry and flowing reaches are attributable to changes in water resources.

We analyze results from a comparison of pitfall trapped arthropods between artificial pools, constructed within the active river channel, and nearby controls, during a drying event, manipulatively testing if previously observed differences in riparian arthropod communities between dry and flowing river sections were caused by differences in water resources. Specifically, we compare dry stream-bed habitats which were near flowing river initially, but which dried by the end of the study, to habitats where we constructed and maintained artificial pools of water as the river dried, supplementing water resources. These pools are unlikely to be a perfect replication of flowing river conditions, but should function to manipulate water resource availability. As a measure of change in community composition, we assessed differences in the Bray-Curtis dissimilarity index applied to changes in biomass or abundance over time. We also examined changes in *α*-diversity and the abundance and biomass of key groups of arthropods suggested by multivariate statistical analyses. Our analyses investigated responses at multiple taxonomic/functional levels including trophic groups, orders, families, and genera within the beetle family Carabidae.

We predicted differential changes in community composition as the river dries, with seasonal increases in diversity at pools only. We also predicted increases in the abundance or biomass of particular carabid beetle genera near pools, as well as increases in other key groups of arthropods, like wolf spiders (Lycosidae), field crickets (Gryllidae), or aerial arthropods. These predictions are based on 1) results from the previous analyses of observational data in this system [39], 2) evidence of partial reliance on surface water of crickets and spiders in this system, based on stable water isotope analysis [39], 3) the effects of changes in riverine resources on riparian arthropods [18], [23], [36], and 4) inference from previous examinations of the influence of flow regime alterations on terrestrial arthropod communities [30]–[32].

## Methods

### Ethics Statement

No specific organization regulates research on invertebrates, but care was taken to minimize unnecessary harm. No species used in this research were considered to be endangered or protected. All necessary permits were obtained for the described field studies. In particular, we received permission from the US Bureau of Land Management and a scientific collecting permit from the State of Arizona, Game and Fish Department (SP736471).

### Study Site

Our study occurred in the active channel along a drying reach of the upper San Pedro River approximately 1.5 km in length near Boquillas Ranch House (31°41′50.95″ N, 110°10′57.15″ W) in the San Pedro Riparian National Conservation Area, managed by the US Bureau of Land Management (BLM). This site is located approximately 15 km downstream from population centers that derive municipal water from the Sierra-Vista sub-watershed groundwater aquifer [6]. Hydrologic studies have linked the aquifer in the region of groundwater pumping to river base flows near our study site [6]. The combination of groundwater pumping with changes in local precipitation regime has led to decreased rates of recharge and may have contributed to recently observed river drying [6], [40]. In the year prior to conducting our research (2005), this reach of river was flowing at the beginning of the spring/summer dry season (April), but dried before the arrival of the summer rainy season (July).

The San Pedro River originates in Mexico and flows north through AZ, USA for 160 km, eventually joining the Gila River, part of the Colorado River drainage [5], [41]. The river floodplain can be quite wide, in places extending up to several hundred meters from the river [42]. Where perennial, this floodplain is often dominated by cottonwood (*Populus fremontii*) and willow (*Salix gooddingii*) trees, but becomes increasingly dominated by tamarisk (*Tamarix* spp.) as groundwater declines and river flows become more intermittent [43]. The uplands near our study site were dominated by plants of Chihuahuan desert. The active river channel was characterized by sand, gravel, and cobble bars, which became exposed at base flow or as the river dried. The river is also temporally variable, with large flood pulses in the summer rainy season (July-September) which can cause substantial disturbance to the floodplain, but which did not occur during our study period.

The San Pedro River has extremely high richness of birds, mammals, and reptiles, and supports endangered species such as the southwest willow flycatcher [5]. Additionally, it is one of the last free-flowing rivers in the western US [41] and may provide an important stopover for migratory birds [44]. Thus, this area is of considerable conservation concern.

### Artificial Pools

**Figure 1:**
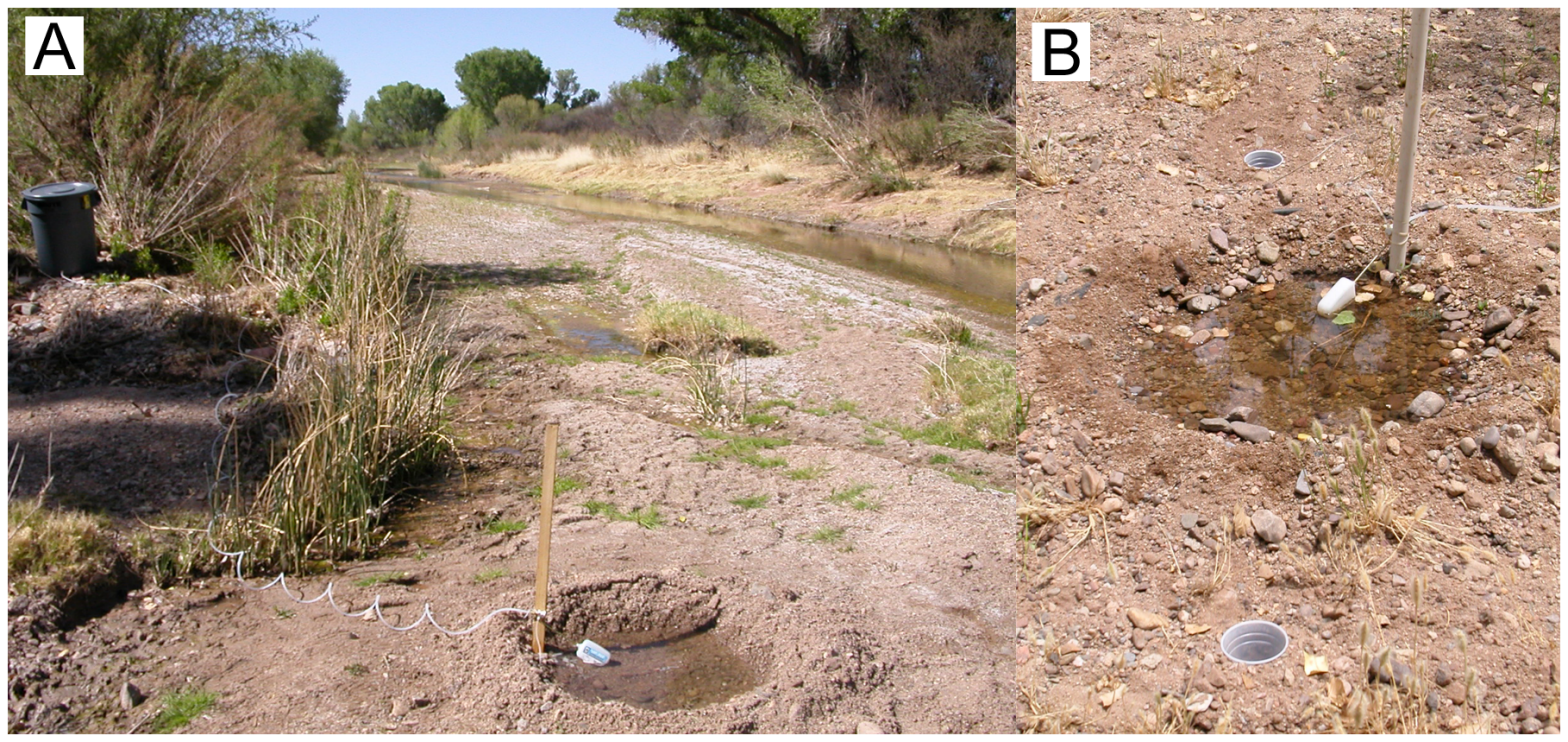
Experimental setup. A. An artificial pool near the beginning of the experiment. B. Pitfall trapping near an artificial pool at the end of the experiment.

In the last two weeks of April 2006, we constructed 10 artificial pools in the active channel of the San Pedro River, within several meters of the flowing river (wetted channel). Longitudinally, pools were spaced ∼150 m apart, with a control site for comparison in between every two pools (∼75 m from either pool), with one extra on the end (10 control sites). Control sites were also initially located in the active channel within several meters of the flowing river (wetted channel). Pools were lined with 114 L (30-gal) Beckett preformed pond liners (Model PP1035) approximately 1 m in diameter and 36 cm deep. Liners were dug into the streambed and refilled with the removed substrate, leaving a slight depression in the middle. Reference control sites were also disturbed by digging, which may or may not have adequately mimicked the disturbance and structure added by the pools. Pools were filled automatically by gravity from nearby tanks (see Text S1, Figure 1). Pools were maintained for approximately 2.5 months as the river dried, until the final sampling on 25 June 2006. Flows continuously declined during this period and rainfall was minimal.

### Flowing Reference Sites

Although this experiment only directly examined differences between pools and dry areas, we also sampled along still flowing sections of the river and report limited information from these sites for reference. At time 0, all sites were flowing but we also marked flowing reference sites approximately 3 km upstream. Since many of these sites dried more quickly than expected we added additional flowing sites upstream as drying progressed. Graphs show comparisons across all sites and dates, but statistical analyses focus on differences on the final sampling date to deal with inconsistent sampling.

### Pitfall Sampling

Sticky pitfall traps were used for all trapping to avoid biases associated with liquid traps across gradients of water availability (K. McCluney, unpublished data, see Text S1 for details). The traps were constructed using 16-oz (473 mL) cups lined with Tangle-trap (The Tanglefoot Company, Grand Rapids, MI) on the bottom 4 cm of the cup and open on the top. Traps were prepared in advance and stored in quart-sized (946 mL) Ziploc bags (see Text S1 for details).

We sampled four times in the late spring and early summer of 2006 (14 May 2006 to 25 June 2006). Every site received two traps, one within 0.5 m to the east of the object of interest (pool, flowing river, dry area) and one within 0.5 m to the west. Cups were buried so that the ground was level with the top of the cup. Traps were immediately open upon placement and left for approximately 24 hours. Traps were removed between sampling events.

Traps were processed by freezing, then soaking with baby oil to dissolve the Tangle-trap followed by filtering (0.5 mm) and collection and identification of anything identifiable as an arthropod with the naked eye. Most arthropods were identified to the family level and all lengths were measured to the nearest 0.5 mm from the tip of the head to the tip of the abdomen. Biomasses of adults were estimated from these measurements using published values for riparian arthropods in California, USA [45]. Direct gravimetric methods were not possible due to residue associated with the pitfall trapping and processing techniques. We also identified all carabids to genus. Identification was aided by Borrer et al. [46], Ubick et al. [47] and Arnett and Thomas [48].

### Other Measurements and Sampling

Aquatic insect samples were collected from pools (still waters) and flowing sites by jab and sweep methods, sweeping a standard aquarium net (∼13×15 cm) three times, gently scraping the bottom. These aquatic samples were frozen until identification. Samples were defrosted and arthropods were picked out of the samples and identified to order or family.

### Data Processing and Statistics

We excluded very small arthropods (less than 1.5 mm) and all collembolans from our pitfall trap data set prior to analyses, due to potential biases in our sample processing procedures. We also excluded crayfish (virile, *Orconectes virilis*, and red swamp, *Procambarus clarkii*) caught in some traps from all analyses and excluded unidentifiable arthropods from community level analyses. We averaged the two traps per site, to produce an estimate of abundance or biomass per trap. Finally, we removed three pool sites and three dry sites, where the river never completely dried, from our primary analyses (see Text S1 for more details).

**Figure 2:**
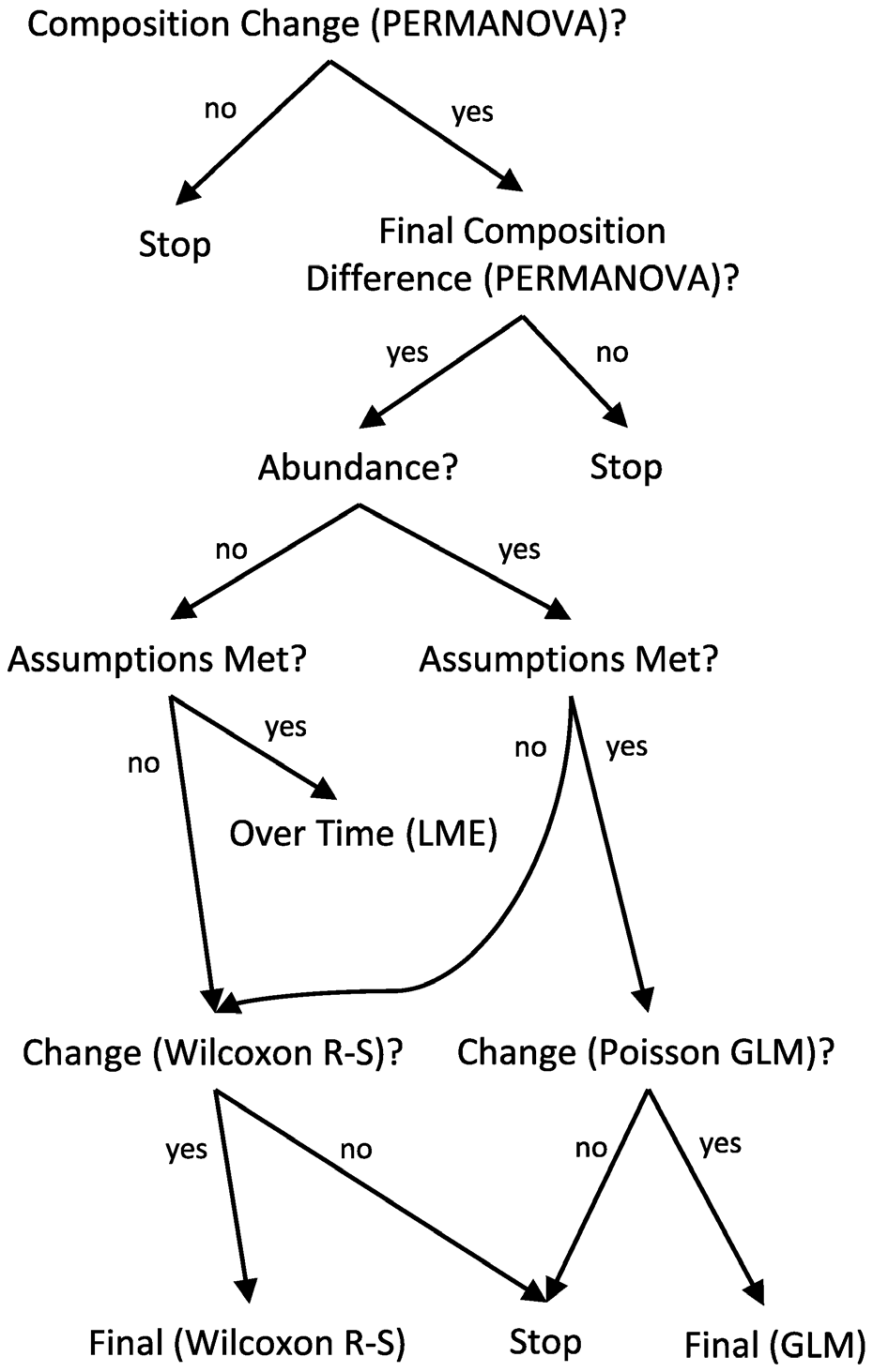
Statistical analysis decision tree.

We employed several statistical methods, all in the statistical program R (v2.15.1). When possible we used likelihood ratio tests of longitudinal linear mixed effects models (LME) of each response metric, with site as a random effect (intercept), and an explicit consideration of multiple plausible temporal variance-covariance structures (compound symmetry, autoregressive, or unstructured). This is an analogous approach to standard RmANOVA, but allows specification of alternative variance-covariance structures. Response metrics analyzed in this manner included diversity and biomass, whenever variance and normality assumptions were met. For some response metrics, this form of analysis was not possible, due to current limitations of longitudinal mixed effects models in R. In particular, different temporal variance-covariance structures cannot currently be specified for tests of multivariate community responses, or for non-gaussian response distributions (e.g., Poisson count data). Therefore, for multivariate community responses, for abundance, and for biomass responses that severely violated assumptions of normality and equal variance (transformations ineffective), we tested for treatment effects on the differences between initial (14 May 2006) and final (25 June 2006) sampling dates. When those temporal difference tests were significant, we tested values from the final date to detect whether responses to treatments diverged or converged. These tests examined whether the response metric changed differentially between treatments across time and whether they converged or diverged, avoiding effects of temporal autocorrelation. However, this approach is less powerful than linear mixed effects modeling. An overview of our analysis approach can be found in Figure 2.

Our first analysis was for changes (final – initial) in community composition over time with permutational multivariate analysis of variance using distance matrices (adonis/PERMANOVA) in the VEGAN package of R v. 2.15.1. These tests took place on transformed data, where we eliminated negative values or those less than 1 by adding a fixed integer and then applied the natural log. Upon finding significant changes in community composition, we followed with a permutational multivariate analalysis of variance on the final date. Whenever we found a significant difference on the final date, we examined non-metric multi-dimensional scaling (nMDS) plots of the community using the VEGAN package of R. Similarity percentages (Simper) analysis was used to identify the most influential taxa, added to NMDS plots.

If community tests suggested both significant differences in how pools and dry sites changed over time and a significant difference in the final communities, we tested for changes in abundance or biomass of individual taxonomic groups. We analyzed differences in changes in abundance and final abundance using generalized linear models (GLM) with either a Poisson or quasi-Poisson distribution, the latter of which helps with modeling overdispersed count data [49]. Since trap counts were averaged at each site, we rounded up to the nearest integer prior to fitting Poisson or quasi-Poisson glm models. For analyses of biomass that met assumptions of normality and equal variance, we conducted likelihood ratio tests for longitudinal mixed models, with time and planned treatment as fixed effects and site as a random effect (intercept), using the lme function in the nlme package in R. Likelihood ratio tests were performed on the change in likelihood when dropping each factor from a model, one at a time, following [50]. When biomass responses were non-normal or had unequal variance, which could not be remedied with transformation, we analyzed only the difference between initial and final and if significant, then the final values using non-parametric Wilcoxon rank-sum tests in R. We followed similar techniques to test for changes in Shannon diversity (H), richness (S), and Pielou's evenness (J), using the VEGAN package and either longitudinal mixed models or glm models, as appropriate.

We compared our artificial pools to flowing sites using 1) aquatic insect samples from a single date, 1 June 2006, 2) multivariate examinations of changes in assemblage composition of aquatic and riparian arthropods, 3) the total abundance and biomass of all pitfall trapped arthropods, and 4) the abundance and biomass of bombardier beetles (Carabidae:*Brachinus*). Due to a lack of dry sites at the beginning of the experiment and the necessity of changing the location of flowing sites throughout the experiment, we analyzed only differences on the final date. To see if bombardier beetles (*Brachinus*), which have ectoparasitic larvae of aquatic dytiscid beetles, were attracted to pools with more dytiscids, we tested for correlations between the two in our pools using Spearman correlations in R v. 2.9.0.

We conducted all relevant analyses at four ecological or taxonomic levels: trophic group, order, family, and genera (only of beetles in the family Carabidae). All of these analyses followed the statistical approaches described above.

Initial nMDS plots revealed that the ground-dwelling arthropod community at one pool site (Pool 3) was very different from all the others for both abundance and biomass and that this site had a large influence on results. Compared to the other pool sites, this site had extremely low abundances of large carabid beetles in the *Chlaenius, Agonum,* and *Brachinus* genera. Additionally, this site seemed to have particularly high abundances of ants on both of the last two dates. Reznikova and Dorosheva [51] found that many carabids tend to avoid high concentrations of ants. Thus, in the body of the paper we report results with this site removed, but report full results in the appendix (Table S1). We also found that one dry site (Dry 9) was very different from other dry sites and had a strong influence on results. Compared to other dry sites, this site had particularly high numbers of carabid beetles in the genus *Brachinus*. Thus we also remove this site from our main analysis, but report results with both sites included in the appendix, for comparison (Table S1). Thus our main analyses were conducted on 6 pools and 6 dry sites and the analyses in the appendix were conducted on 7 pools and 7 dry sites. Comparisons of pools to flowing sites on the final date were conducted on 4 flowing sites and 10 pools, and comparisons of aquatic insect abundance on 1 Jun 2006 were on 3 flowing sites and 7 pools.

## Results

### Total abundance

We did not observe a significant change in total abundance of all pitfall-trapped arthropods between artificial pools and control sites that dried (χ^2^ = 0.05, df = 1, p = 0.826, Table S1).

### Trophic groups

**Figure 3:**
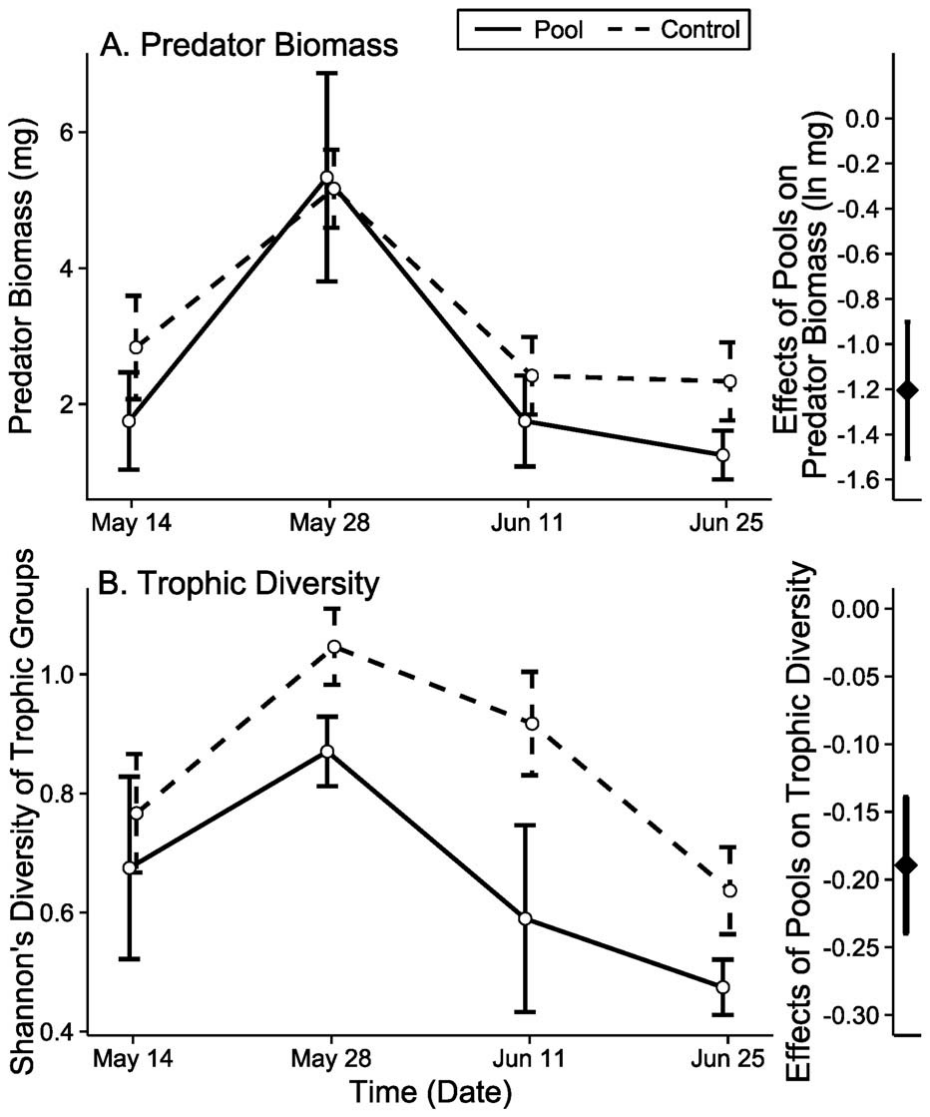
Trophic group responses to experimental treatments. Predator biomass (A) and trophic group diversity (B) of pitfall-trapped arthropods were significantly and consistently higher at control sites that dried than at artificial pools (no time x treatment, Table 1). Fig. S1 and S2 show similar patterns for trophic group richness and evenness. Error bars are standard error. “Effect” of pools on each response is the difference in parameter estimates from mixed effects modeling.

We found no significant difference in the change in the assemblage of trophic groups between artificial pools and control sites that dried (PERMANOVA Abundance: F_1,11_ = 0.64, p = 0.571; PERMANOVA Biomass: F_1,11_ = 1.67, p = 0.070, Table S1). Despite the lack of a detected effect on trophic group composition, we examined changes in abundance and biomass of predators and diversity of trophic groups in order to make comparisons with a previous observational study [22] that found more predators along flowing than dry sections of this river. In this study, we found no treatment effect on abundance (Poisson GLM: χ^2^ = 0.02, df = 1, p = 0.889, Table S1), but we found slightly higher predator biomass at control sites than at artificial pools (LME: χ^2^ = 10.07, df = 1, p = 0.002, Table 1; Figure 3A). We also found higher Shannon’s diversity, richness, and Pielou’s evenness of trophic groups at control sites than at artificial pools (Shannon’s diversity LME: χ^2^ = 9.33, df = 1, p = 0.002, Richness LME: χ^2^ = 7.77, df = 1, p = 0.005, Pielou’s evenness LME: χ^2^ = 6.72, df = 1, p = 0.010, Table 1; Figures 3B, S1, & S2). All of these differences were consistent across time (no time x treatment interaction, Table 1), suggesting that control sites started out with higher predator biomass and trophic diversity and maintained it across time.

**Table 1:** Results of likelihood ratio tests for the effect of removing each fixed effect term from a full longitudinal linear mixed effects model (following Bolker et al [50]).

Model component removed (Fixed Effects)	df	ΔAIC	LRT (χ^2^)	p-value
Predator Biomass				
-Time * treatment	1	−1.95	0.05	0.816
-Time	1	−1.89	0.11	0.738
*-Treatment*	*1*	*8.08*	*10.07*	***0.002***
Trophic Group Shannon’s Diversity				
-Time * treatment	1	−1.75	0.25	0.616
-Time	1	1.56	3.56	0.059
*-Treatment*	*1*	*7.33*	*9.33*	***0.002***
Trophic Group Pielou’s Evenness				
-Time * treatment	1	−1.87	0.12	0.720
*-Time*	*1*	*6.32*	*8.31*	***0.004***
*-Treatment*	*1*	*4.72*	*6.72*	***0.010***
Trophic Group Richness				
-Time * treatment	1	−1.92	0.08	0.784
*-Time*	*1*	*2.80*	*4.80*	***0.028***
*-Treatment*	*1*	*5.77*	*7.77*	***0.005***
Coleoptera Biomass				
*-Time* * *treatment*	*1*	*6.26*	*8.25*	***0.004***
Order Shannon’s Diversity				
*-Time* * *treatment*	*1*	*3.00*	*5.11*	***0.025***
Carabidae Biomass				
*-Time* * *treatment*	*1*	*5.68*	*7.68*	***0.006***
Lycosidae Biomas				
-Time * treatment	1	−1.92	0.08	0.776
-Time	1	−1.87	0.13	0.723
*-Treatment*	*1*	*7.53*	*9.52*	***0.002***
Family Shannon’s Diversity				
-Time * treatment	1	−1.70	0.30	0.582
-Time	1	−1.53	0.47	0.492
*-Treatment*	*1*	*4.00*	*6.00*	***0.014***
Family Pielou’s Evenness				
-Time * treatment	1	−1.96	0.04	0.848
-Time	1	0.60	2.60	0.107
*-Treatment*	*1*	*5.56*	*7.56*	***0.006***
Brachinus Biomas				
*-Time* * *treatment*	*1*	*4.33*	*6.33*	***0.012***
Carabid Genera Richness				
*-Time* * *treatment*	*1*	*2.34*	*4.34*	***0.037***

*df = degrees freedom, ΔAIC = change in AIC associated with removal of each model term, LRT (χ^2^) = the χ^2^ test statistic associated with the change in likelihood with removal of each model term. All models share a single random effect of trap location (example specification: *lme(log(Pred.bio+1)* ∼ samp.day*Treatment, random = ∼*1* | *Location, data = PoolCompAll.d.r, correlation = corCompSymm(form = *∼*1* | *Location), method = “ML”)*). Only responses with a treatment effect are shown here.

### Orders

**Figure 4:**
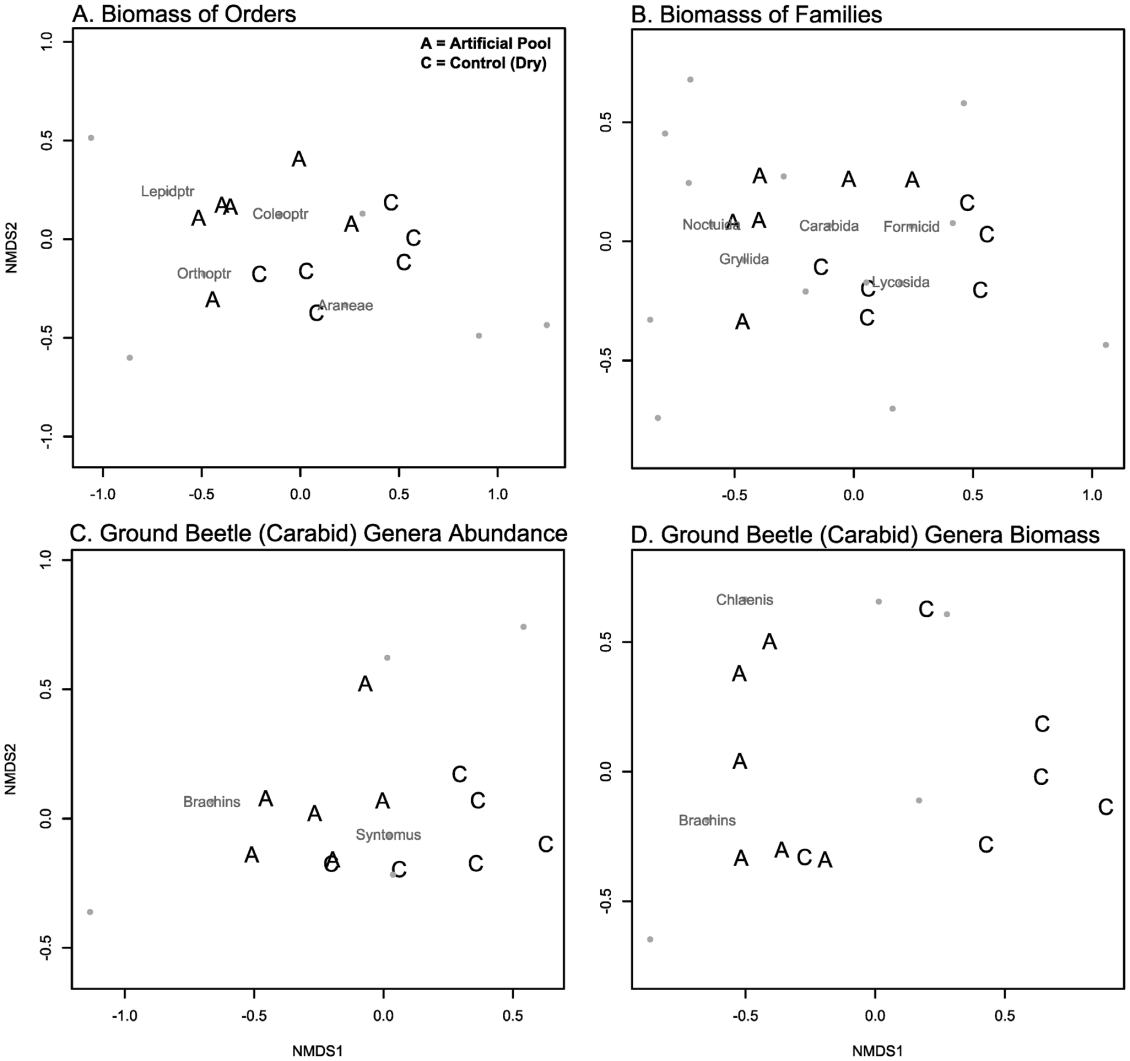
Assemblage differences on the final sampling date, between artificial pools and control sites that dried. Each plot is a non-metric multidimensional scaling ordination, with the letters “A” denoting artificial pool sites, “C” control sites, and with shortened taxa names added for the most influential taxa according to Simper analysis. Other taxa are shown as grey dots, but are not labelled. Plot A and B are the biomass of pitfall-trapped arthropod orders and families, respectively, per trap. Plots C and D are the abundance and biomass of pitfall-trapped carabid genera, respectively, per trap. Both the change over time (not shown, PERMANOVA Orders: F_1,11_ = 2.52, p = 0.001, PERMANOVA Families: F_1,11_ = 2.64, p = 0.002, PERMANOVA Carabid Abundance: F_1,11_ = 4.23, p = 0.012, PERMANOVA Carabid Biomass: F_1,11_ = 3.01, p = 0.003, Table S1) and the final assemblage composition (shown, PERMANOVA Orders: F_1,11_ = 4.66, p = 0.025, PERMANOVA Families: F_1,11_ = 3.40, p = 0.030, PERMANOVA Carabid Abundance: F_1,11_ = 5.69, p = 0.012, PERMANOVA Carabid Biomass: F_1,11_ = 7.81, p = 0.005, Table S1) were significantly different between artificial pools and control sites that dried.

**Figure 5:**
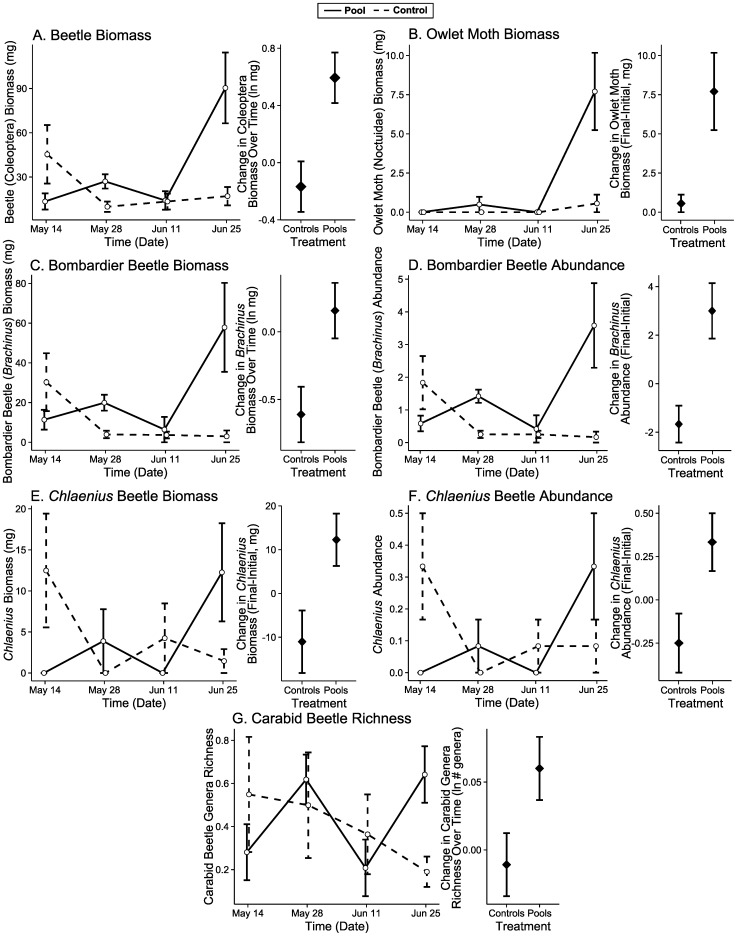
Pitfall trapped arthropods with positive responses to artificial pools. Beetle (A, Table 1) and owlet moth (B, Table S1) biomass increased at artificial pools, but either declined or did not increase at control sites that dried. The biomass (C, E, Table 1) and abundance (D, F, Table S1) of two genera of ground beetles (*Brachinus*, C&D, *Chlaenius*, E&F) had a similar response to overall beetle biomass. The richness of genera of carabid beetles also increased at pools, but declined at control sites as they dried (G). Error bars are standard error. “Change” of each response over time is derived from parameter estimates from mixed effects modeling.

**Figure 6:**
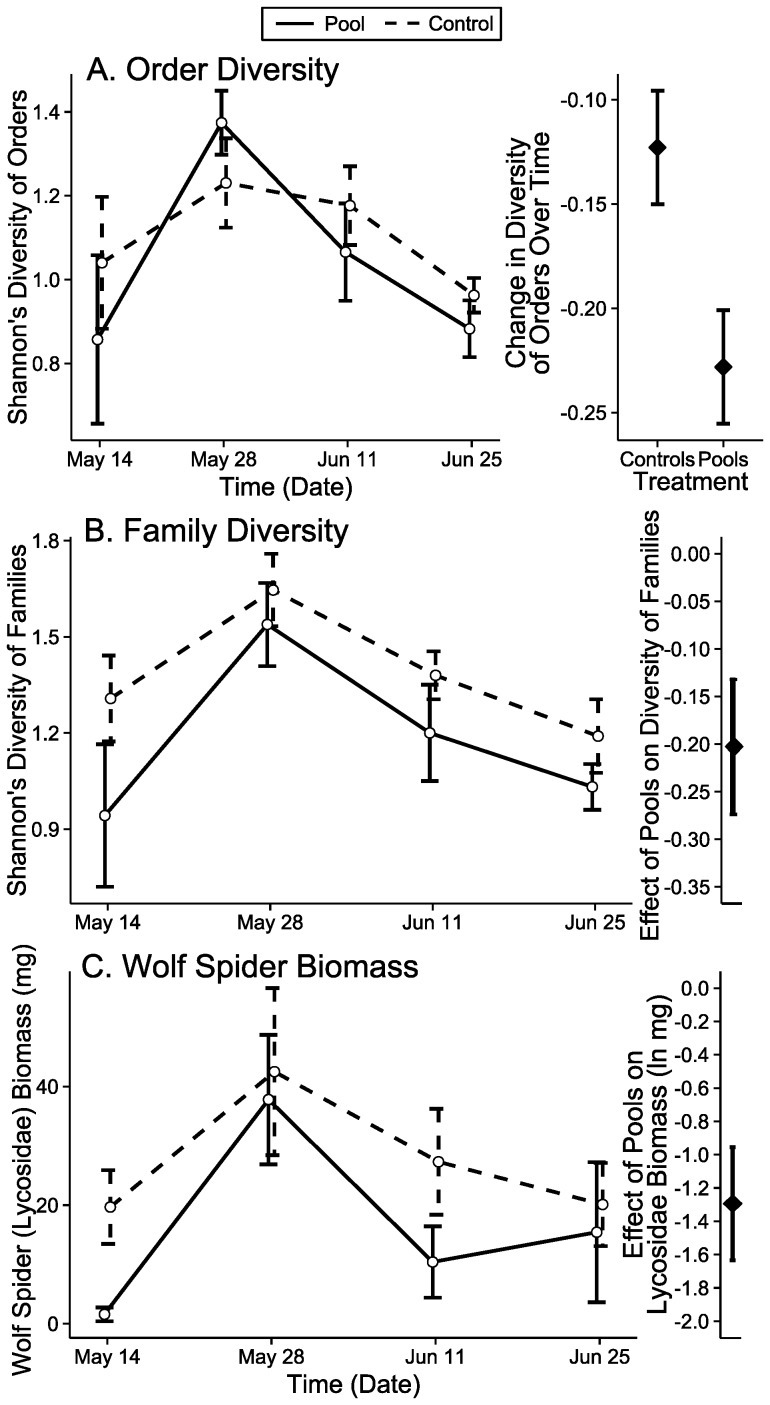
Pitfall trapped arthropods higher at control sites. Order diversity (A), family diversity (B), and wolf spider biomass (C) were all consistently higher at control sites across the experiment (Table 1). This matched patterns of predator biomass and diversity of trophic groups (Figure 3). Error bars are standard error. “Change” in the response of order diversity over time is derived from parameter estimates from mixed effects modeling. “Effect” of pools on each response is the difference in parameter estimates from mixed effects modeling.

The assemblage of orders of arthropods changed between the initial and final dates differentially for artificial pools and controls that dried (PERMANOVA Biomass: F_1,11_ = 2.52, p = 0.001). The final date showed differences between these sites, showing evidence of divergence (PERMANOVA: F_1,11_ = 4.66, p = 0.025, Figure 4A). We only found a significant contribution of beetles (Coleoptera) to changes at these sites (LME: χ^2^ = 8.25, df = 1, p = 0.004, Table 1, Figure 5A), with an increase at artificial pools and a decrease at control sites that dried. We also found a significant time by treatment interactive effect on Shannon’s diversity of orders, with a complicated pattern over time, but overall a greater decline in diversity at pool sites than at control sites (LME: χ^2^ = 5.00, df = 1, p = 0.025, Figure 6A). We note that in each analysis we report, here and below, a lack of a detectable univariate response for any particular taxon does not mean that this taxon was not influential. Differences in multiple rare taxa could have resulted in multivariate differences between sites, but changes in abundance or biomass individually could be undetectable due to low sampling. Non-metric multi-dimensional scaling graphs and Simper analysis provide clues to other taxa that may have influenced community level differences. Lepidoptera, Orthoptera, and Araneae may also have played important roles in the community level differences between pools and dry sites (Figure 4A).

### Families

The assemblage of families of arthropods changed between the initial and final dates differentially for pools and controls that dried (PERMANOVA Biomass: F_1,11_ = 2.64, p = 0.002). The final date showed differences between these sites, showing evidence of divergence (PERMANOVA Biomass: F_1,11_ = 3.40, p = 0.030, Figure 4B). Carabid ground beetles (Carabidae; LME: χ^2^ = 7.68, df = 1, p = 0.006, Table 1, Figure S3), wolf spiders (Lycosidae; LME: χ^2^ = 9.52, df = 1, p = 0.002, Table 1, Figure 6C), and owlet moths (Noctuidae; Wilcoxon R–S: W = 31.5, p = 0.026, Figure 5B, Table S1) contributed significantly to these differences. Carabids and owlet moths increased in biomass at artificial pools, while wolf spiders had consistently higher biomass at control sites, from the beginning of the experiment. Simper analysis suggested that field crickets (Gryllidae) and ants (Formicidae) may also have been important in community differences, but univariate analyses failed to show a response. Shannon’s diversity and Pielou’s evenness were consistently higher at control sites than at artificial pools, from the beginning of the experiment to the end (LME Shannon’s diversity: χ^2^ = 6.00, df = 1, p = 0.014, Figure 6B, Pielou’s evenness:, Table 1, Figure S4).

### Carabid genera

The assemblage of carabid ground beetles changed differentially between artificial pools and controls sites that dried (PERMANOVA Abundance: F_1,11_ = 4.23, p = 0.012, PERMANOVA Biomass: F_1,11_ = 3.01, p = 0.003). Differences between treatments on the final date suggested divergence (PERMANOVA Abundance: F_1,11_ = 5.69, p = 0.012, Figure 4C, PERMANOVA Biomass: F_1,11_ = 7.81, p = 0.005, Figure 4D). The genera *Brachinus* (bombardier beetles) and *Chlaenius* contributed significantly to the change in relative abundance and biomass, with increases at pools, but decreases at control sites that dried (LME *Brachinus* Biomass: χ^2^ = 6.33, df = 1, p = 0.012, Figure 5C, Table 1, Wilcoxon R–S Change in *Chlaenius* Biomass: W = 31.5 p = 0.026, Wilcoxon R–S Final *Chlaenius* Biomass: W = 25.5, p = 0.182, Figure 5E, Table S1, GLM Change in *Brachinus* Abundance: χ^2^ = 13.41, df = 1, p<0.001, GLM Final *Brachinus* Abundance: χ^2^ = 23.66, df = 1, p<0.001, Figure 5D, GLM Change in *Chlaenius* Abundance: χ^2^ = 1.97, df = 1, p = 0.010, GLM Final *Chlaenius* Abundance: χ^2^ = 1.05, df = 1, p = 0.253, Figure 5F, Table S1). Simper analysis suggested the genus *Syntomus* may have also contributed to differences in abundance. The richness of Carabidae genera increased significantly more at artificial pool sites than at controls that dried (LME: χ^2^ = 2.34, df = 1, p = 0.037, Figure 5G, Table 1). There was no significant correlation between bombardier beetles (*Brachinus*) and diving beetles (Dytiscidae) in our pools (Spearman: r = 0.72, S = 9.86, n = 6, p = 0.11).

### Comparison to flowing reference sites

**Figure 7:**
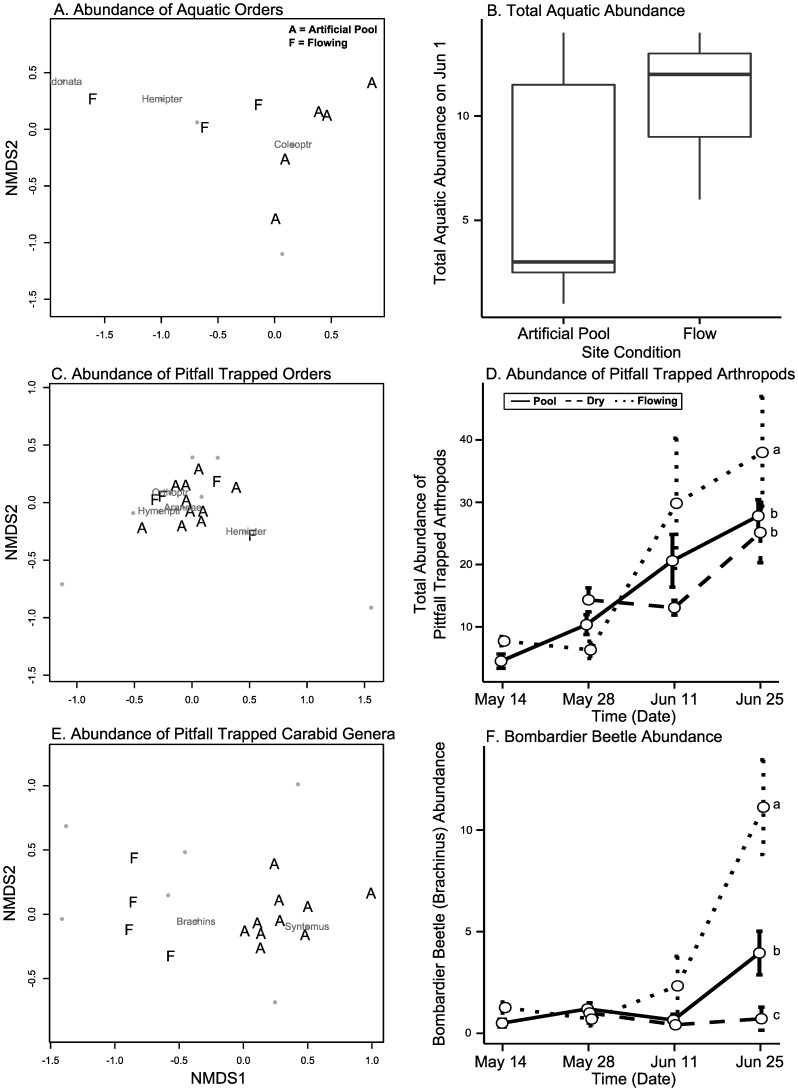
Comparisons of artificial pools to flowing reference sites. Plots A, C, and E show significant differences (Table S2) between assemblage composition at artificial pool (“A”) and flowing (“F”) sites using nonmetric multidimensional scaling plots of the abundance of orders of aquatic insects (plot A), pitfall trapped arthropod orders (plot C), and pitfall trapped carabid genera (plot E). Plots B, D, and F show significant differences in the total abundance of aquatic insects (plot B), all pitfall trapped terrestrial arthropods (plot D), and bombardier beetles (*Brachinus*, plot F, Table S2). Plot B is a standard box plot and error bars in plots D and F are standard error. In plots D and F, small letters denote significant pairwise Tukey’s differences between site conditions on the final date.

The assemblage of orders of aquatic insects differed between artificial pools and flowing sites on 1 Jun 2006 (PERMANOVA Abundance: F_1,9_ = 7.57, p = 0.009, Table S2, Figure 7A), with Odonata, Hemiptera, and Coleoptera most influential according to Simper analysis. Total abundance of all aquatic insects was higher at flowing sites (GLM: χ^2^ = 4.26, df = 1, p = 0.039, Figure 7B). The composition and relative abundance of orders of pitfall trapped arthropods also differed significantly between artificial pools and flowing sites on the final sampling date (PERMANOVA Abundance: F_1,13_ = 2.38, p = 0.035, Figure 7C), as did genera of carabid beetles (PERMANOVA Abundance: F_1,13_ = 20.41, p = 0.001, Figure 7E). However we did not find a difference in the composition and relative abundance of pitfall-trapped families between these sites (PERMANOVA Abundance: F_1,13_ = 1.37, p = 0.216). On the final sampling date, the total abundance (GLM: χ^2^ = 14.13, df = 2, p = 0.001) and biomass (GLM: F_2,18_ = 6.17, p = 0.009) of all pitfall trapped arthropods was higher at flowing sites than at either dry (Tukey’s: Abundance p<0.001, Biomass p = 0.001) or artificial pool sites (Tukey’s: Abundance p = 0.007, Biomass p = 0.038), which were equivalent (Tukey’s: Abundance p = 0.525, Biomass p = 0.272, Figure 7D). On the final sampling date, bombardier beetle abundance (GLM: χ^2^ = 63.30, df = 2, p<0.001) differed significantly between dry, artificial pool, and flowing sites, with flowing sites having highest abundance, artificial pools intermediate abundance, and dry sites the lowest abundance (Tukey’s: Flowing-Dry p<0.001, Flowing-Pool p<0.001, Dry-Pool p<0.001, Figure 7F). Biomass (GLM: F_2,18_ = 9.55, p = 0.001) was similar, except that we did not detect differences between artificial pools and flowing sites (Tukey’s: Flowing-Dry p<0.001, Flowing-Pool p = 0.230, Dry-Pool p = 0.003).

## Discussion

Groundwater pumping, climate change, and regional droughts can alter surface flows in rivers, but the effects of river drying on riparian animal communities have not been well studied. We document several effects of altered water resources on riparian communities: 1) shifts in community composition, 2) changes in richness and diversity at certain taxonomic levels and 3) decreased abundance and/or biomass of some groups of arthropods, including carabid beetles and owlet moths. Our results suggest that water resource availability, movement and colonization dynamics, and tolerance mechanisms interact to influence patterns of arthropod community structure and diversity along drying rivers.

The current study supports a mechanistic role for water resources in some of the patterns observed in a previous observational study along this section of river [22]. In particular, the abundance of carabid beetles in the genera Brachinus (bombardier beetles) and Chlaenius, as well as the overall richness of carabid beetle genera increased at artificial pools, but declined at control sites that dried. These two genera drove similar patterns found in the biomass of ground beetles (Carabidae) and beetles more generally (Coleoptera). Thus, water resources seem to have important effects on beetles, particularly two genera of carabids.

We found several surprising differences between our current water resource manipulation using pools and the previous comparison of flowing and dry sites. Most notably, artificial pools had lower diversity of trophic groups, orders, and families, as well as lower biomasses of predators, particularly wolf spiders. This contrasts with the finding from the previous study that flowing sites had higher diversity and higher predator and wolf spider abundance. We hypothesize that these differences could be due to several important interacting factors. First, artificial pools were newly constructed water sources, as opposed to flowing river sites. Thus, colonization dynamics likely played a role. More time may have been needed for the communities at these artificial pools to reach an equilibrium. Second, while many arthropods probably migrate in response to river drying, some may be less prone, *sensu* [52]. Predators, particularly wolf spiders, maintained high biomass at control sites, even as they dried. Our samples were dominated by the beach wolf spider (*Arctosa littoralis*), a long-lived, large-bodied species capable of digging burrows. We suggest that due to the likely ability of this species to reduce water demands with burrows [53] and to meet water demands by consuming moist prey [23] (Figure S1), *A. littoralis* may have reduced rates of migration compared with other species. Both through positive effects of predation on diversity [54], [55] and the potential for other rare species to exhibit similar river drying tolerant behavior, overall diversity could remain high at dry sites for a short time as a legacy of previous flows. Pools, on the other hand, were constructed at least a meter or two from the river and may not have started out with quite the same community as the control sites. We suggest that the low diversity at pools was likely due to the combined factors of incomplete colonization near pools and of philopatry to previously flowing areas by some species that had behavioral adaptations to drying. However, long-term equilibrium may differ and even over the short term, diversity was less at dry sites than flowing reference sites [22]. In general, the patterns of diversity and community dynamics found in this study are consistent with metacommunity theory, where diversity patterns are driven by the interplay of dispersal and species interactions [56]. Our findings agree with evidence of differential sensitivity and resilience of aquatic species to river drying events [7], [12], [17], but these terrestrial communities may be less sensitive, with some species able to persist on terrestrial resources alone.

Both owlet moths and some genera within the family Carabidae (e.g., *Brachinus*) responded particularly strongly and positively to pools. These taxa are mobile and known to be tied to water resources. The positive response of *Brachinus* to pools may be due to 1) attraction to aquatic beetles (e.g. Dytiscidae) that serve as hosts for ecto-parasitic larvae of *Brachinus* [57], [58], 2) attraction to emergent aquatic insects as a source of food [58], 3) attraction to terrestrial prey such as aerial insects that had greater biomass near pools [58], or 4) attraction to increased water availability [59]. On 1 June 2006, we found significantly higher total abundance of aquatic insects at flowing reference sites than at our pools, as well as differences in community composition. We also tested for correlations between *Brachinus* and Dytiscidae abundance in artificial pools on this date, but found no significant correlation. At the final sampling, the mean abundance of *Brachinus* at flowing reference sites was greater than that at our artificial pools (Figure 7f). This suggests that our pools had some positive effects on *Brachinus*, but did not completely replicate flowing river resources.

Owlet moths have been found in other southwestern riparian areas [60] and many species of Lepidoptera have been observed *puddling* (aggregating near puddles) in a variety of other studies [61] and in this study (K. McCluney, personal observations; Figure S2). In addition to attraction to water resources, it is also thought that Lepidoptera that exhibit puddling behavior and drinking may be seeking sodium ions [62]. Thus, pools may provide multiple resources to these animals. While Noctuidae and other aerial insects may not reside near pools, their mobility may allow them to regularly visit nearby water sources, behaviorally responding to changes in water availability more quickly than ground-dwelling arthropods.

In addition to owlet moths, other Lepidoptera and winged Hymenoptera were observed to regularly visit artificial pools to drink (K. McCluney, personal observation; Figure S3). Of the Hymenoptera, bees and parasitic wasps seemed particularly frequent visitors to pools (e.g., photograph of a tarantula hawk, family Pompilidae, drinking, Figure S7). These aerial taxa were rarely collected in pitfall traps, and thus we may have missed one factor influencing ground arthropod communities. This may have contributed to the lower biomass of wolf spiders (Lycosidae) at pools than at dry sites. Increased visits to pools by parasitic spider wasps (Pompilidae) could have caused decreases in large spiders through parasitism or through behavioral avoidance of pools by spiders. Unfortunately, we poorly sampled strong fliers like spider wasps, so we cannot test this hypothesis here.

Other aerial consumers also used our pools. Motion-activated cameras recorded several omnivorous, insect-eating bird species drinking from these pools (bird species: house finch, lesser goldfinch, lazuli bunting, song sparrow, Figure S8). Toads were also found in and near pools (Figure S9). Thus, pools attracted a variety of insectivorous predators and could have reduced the potential positive effects of pools on arthropods. Temporary pools of water may represent a source of limited resources, but may also present great risk of predation and thus these pools may be involved in foraging games surrounding water resources [63].

Despite previous work in our study system that pinpoints water as a resource responsible for structuring communities [23], [42], the role of energy and nutrients cannot be dismissed. The importance of riverine subsidies of energy and nutrients has recently received great attention [18]–[21], [28], [36], [64], [65]. In our study, aquatic insect abundances were lower at pools than at flowing reference sites, suggesting possible energetic components to the differences in terrestrial arthropods between pools and flowing sites. However, the response of some taxa (e.g. noctuid moths) was probably not influenced by aquatic insects.

As rivers dry, many riparian consumers may be forced to meet water demands solely by consuming moist food [23], [42]. Aquatic insects are not only a source of energy and nutrients, but also a source of water (trophic and metabolic) to terrestrial predators. Thus, while decreases in energy or nutrient subsidies associated with river drying may have contributed to our observations, meeting water demands may be a more immediate limitation [23], [66], playing a role in the response to drying.

### Caveats

We note that we did not detect significant differences for many of the responses previously reported to differ between flowing and dry sites along this river [22]. This lack of detection could be due to real differences between flowing sites and pools, as reported here, but low sample sizes in this intensive manipulative experiment could have also contributed. Additionally, many taxa had low collection frequencies and zeros, which limited our ability to detect changes. With greater capture levels or replication, we may have had greater ability to discern patterns statistically.

Another factor potentially influencing our results is the location of our experiments. Pools and traps were located along the active channel of the river, in areas with high concentrations of gravel, sand, and cobble bars and little vegetation. Floodplains in this system tend to be leaf litter dominated with an overstory of cottonwood and willow trees and may harbor different species in different abundances. For instance, field crickets (Gryllidae) appear to be more abundant in floodplains (K. McCluney, personal observations). Also, while the large, beach wolf spider, *Arctosa littoralis*, is often found in river channels, it is rarely found in floodplains, instead being replaced by the large wolf spider *Hogna antelucana* (K. McCluney, personal observations). Thus, by sampling the active channel, we may have missed sampling key areas of abundance for certain taxa, such as Gryllidae, and thus had insufficient capture rates of these taxa to observe significant differences in univariate analyses.

### Broader implications of river drying for riparian communities

It is important to note that this particular reach of the San Pedro River currently dries for only 1–2 months per year and the cottonwood-willow forest of the floodplain is abundant. Thus, we examined only short-term effects of river drying. Other studies have shown substantial changes in vegetation composition, structure, and abundance in other sections of this river that dry more frequently and in years when groundwater levels drop [13], [14], [67]. Further Sabo et al [42] and McCluney and Sabo [23] suggest that the cricket *Gryllus alogus* (the most abundant species of Gryllidae in our samples) may depend on greenfall from these forests to meet water demands. Thus, long-term changes in the plant community associated with river drying may result in more dramatic changes to the ground-dwelling arthropod community.

Overall, our research suggests that in the short-term, river drying and temporary pools change the riparian arthropod community via negative effects of declining water resources on noctuid moths and carabid beetles. However, given the dynamic nature of these systems, there may be some resilience to short-term drying events. Effects of more severe dewatering and increased frequency or duration of river drying may differ.

Due to their dynamic nature and high concentrations of resources, riparian areas are often essential for animal communities, especially in dryland climates like southeastern AZ, USA [44], [68], [69]. Further, riparian animals provide important ecosystem services (e.g. recreational bird watching) with both clear direct and indirect monetary values [29]. Thus, streamside areas are of critical conservation concern. We know from extensive previous research that drastic changes to flow regimes can greatly alter riparian vegetation [5], [14]. However, here we show that even short drying events may have direct effects on terrestrial animal communities through alteration of water resources, but some degree of resistance and resilience is clear. Managing rivers for the benefit of multiple users will require incorporating an understanding of the effects of river drying on terrestrial animal communities.

## Supporting Information

Figure S1 **Richness of trophic groups of pitfall-trapped arthropods.** Error bars are SE. See Table 1.
(TIF)

Figure S2 **Evenness of trophic groups of pitfall-trapped arthropods.** Error bars are SE. See Table 1.
(TIF)

Figure S3 **Biomass of carabid beetles.** Error bars are SE. See Table 1.
(TIF)

Figure S4 **Pielou’s evenness of families of pitfall-trapped arthropods.** Error bars are SE. See Table 1.
(TIF)

Figure S5 **The beach wolf spider (** ***Arctosa littoralis*** **) consuming an adult female damp-loving field cricket (** ***Gryllus alogus*** **) along a dry section of the San Pedro River, near the study site.**
(TIF)

Figure S6 **Lepidoptera puddling at an artificial pool.**
(TIF)

Figure S7 **Bees and a tarantula hawk (family Pompillidae) drinking from an artificial pool.**
(TIF)

Figure S8 **Omnivorous insect-eating birds drinking from pools.** Song sparrow, house finch, lesser goldfinch, lazuli bunting.
(TIF)

Figure S9 **A toad using one of the artificial pools.**
(TIF)

Table S1 **Comparison of results with and without including 2 sites (one pool and one dry) with uncharacteristic distributions of carabid beetles and/or ants.**
(DOCX)

Table S2 **Results of tests comparing artificial pools to flowing reference sites.**
(DOCX)

Text S1 **Supplementary Methods.**
(DOC)
