# Supplementary figures and images for: Sensitivity and Tolerance of Riparian Arthropod Communities to Altered Water Resources along a Drying River
Source: PLoS One. 2014 Oct 8;9(10):e109276. doi: 10.1371/journal.pone.0109276 (PMC4190312; doi:10.1371/journal.pone.0109276)

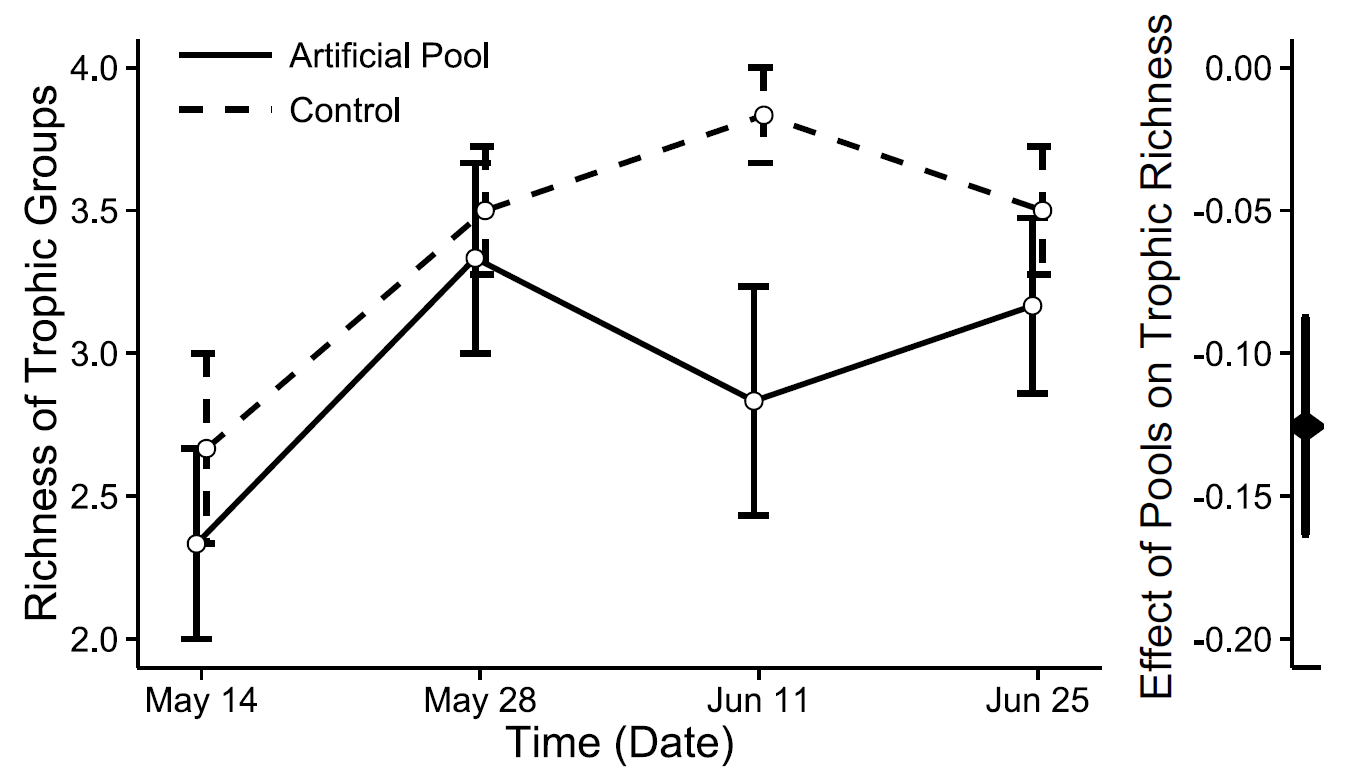

Supplement: Figure S1 — Richness of trophic groups of pitfall-trapped arthropods. Error bars are SE. See Table 1. (TIF) [file pone.0109276.s001.tif]

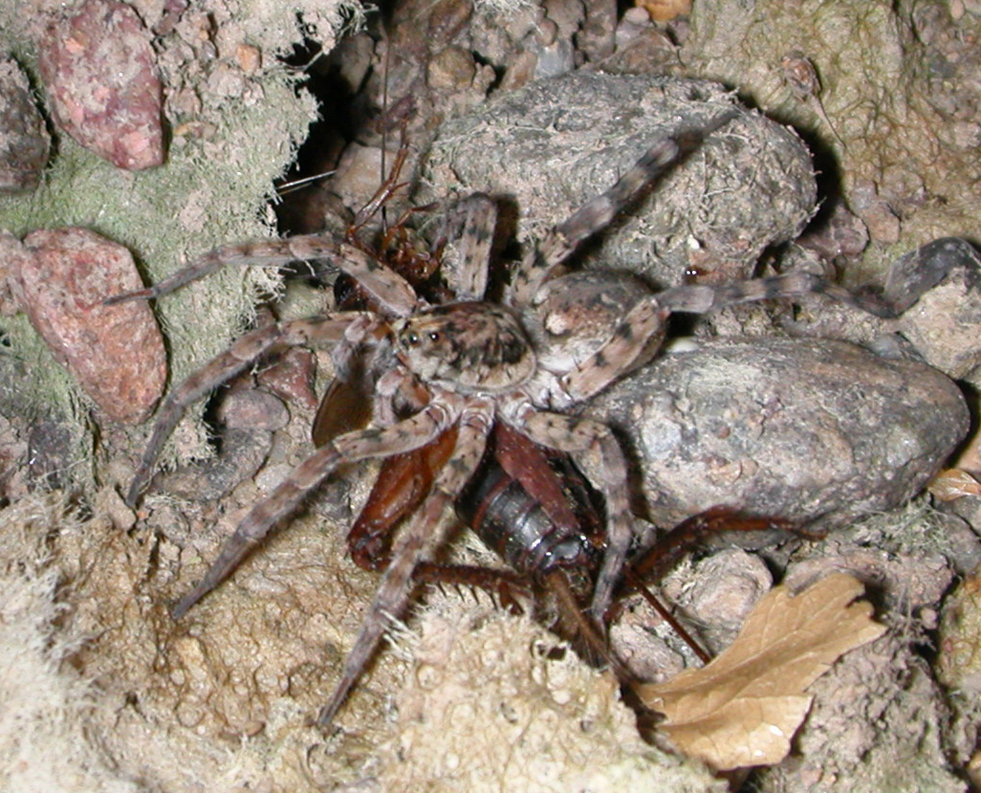

Supplement: Figure S5 — The beach wolf spider ( Arctosa littoralis ) consuming an adult female damp-loving field cricket ( Gryllus alogus ) along a dry section of the San Pedro River, near the study site. (TIF) [file pone.0109276.s005.tif]

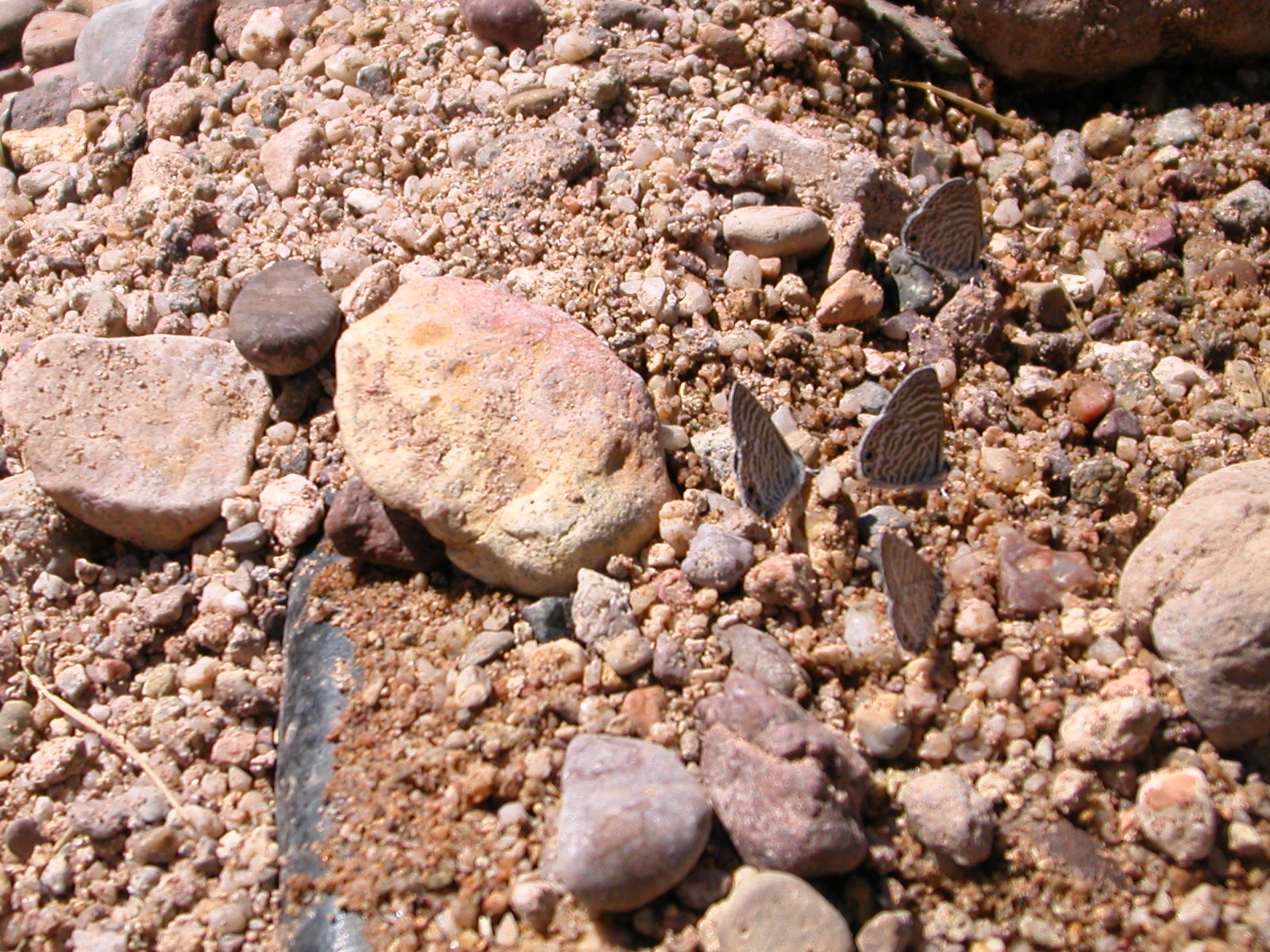

Supplement: Figure S6 — Lepidoptera puddling at an artificial pool. (TIF) [file pone.0109276.s006.tif]

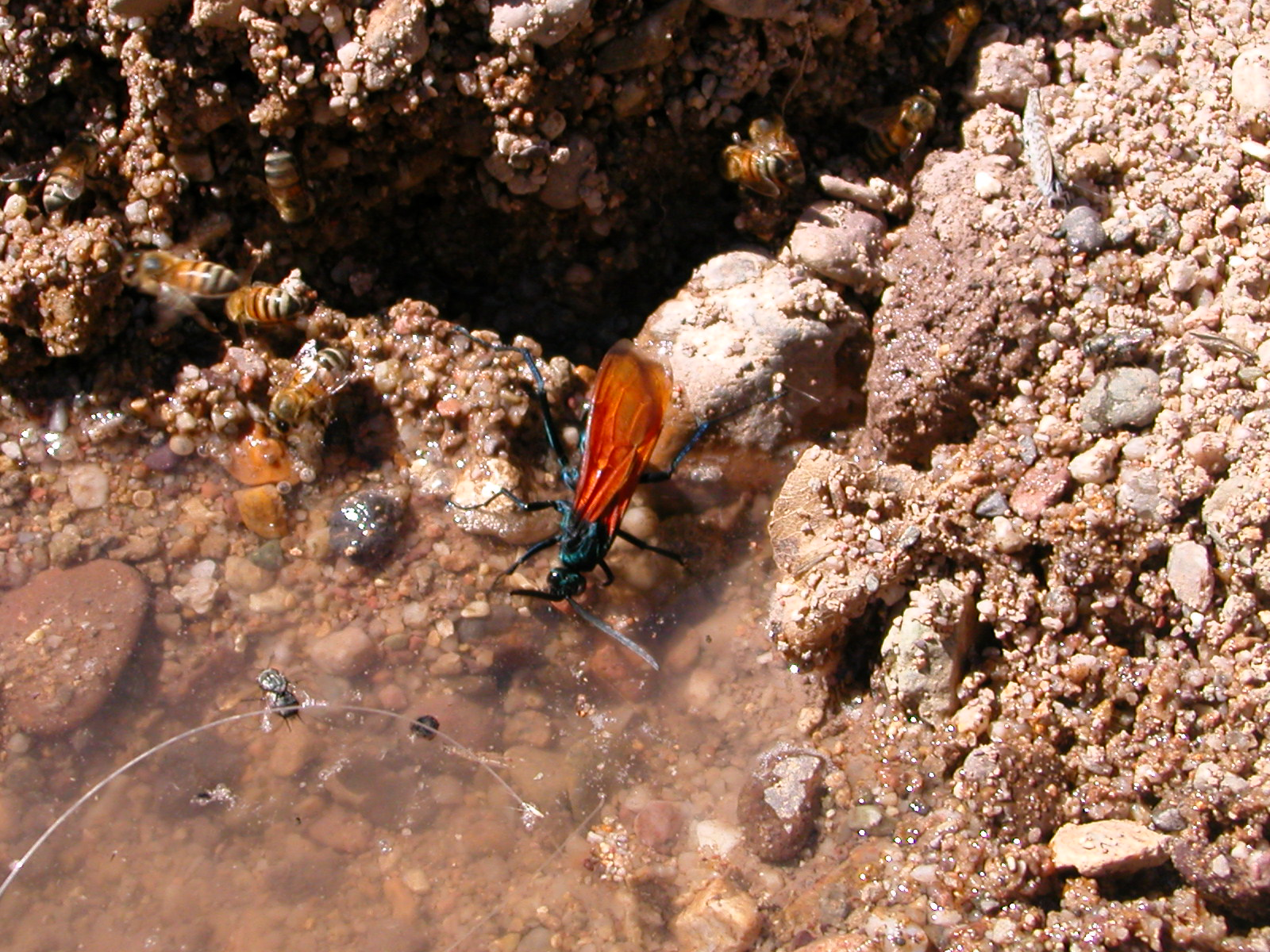

Supplement: Figure S7 — Bees and a tarantula hawk (family Pompillidae) drinking from an artificial pool. (TIF) [file pone.0109276.s007.tif]

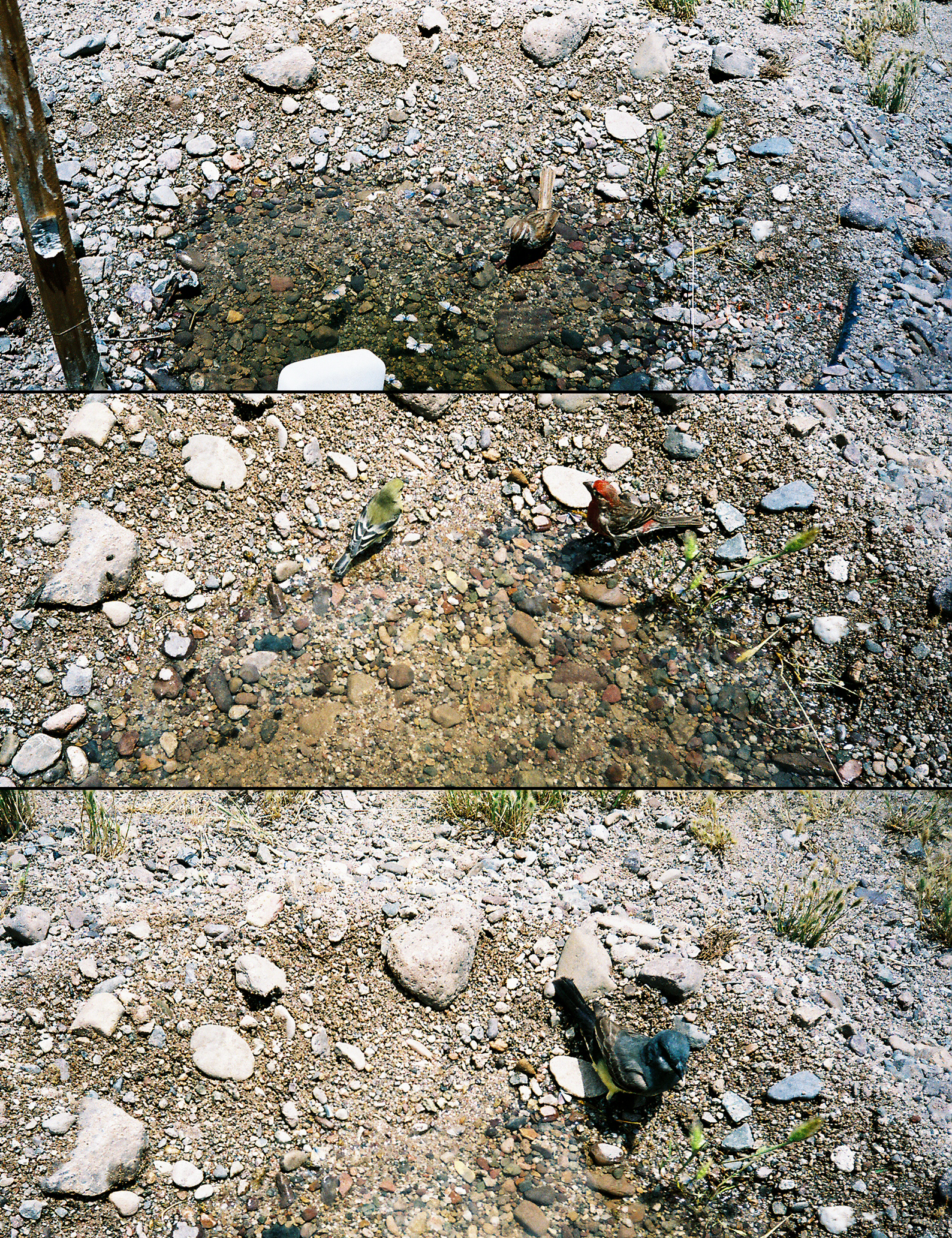

Supplement: Figure S8 — Omnivorous insect-eating birds drinking from pools. Song sparrow, house finch, lesser goldfinch, lazuli bunting. (TIF) [file pone.0109276.s008.tif]

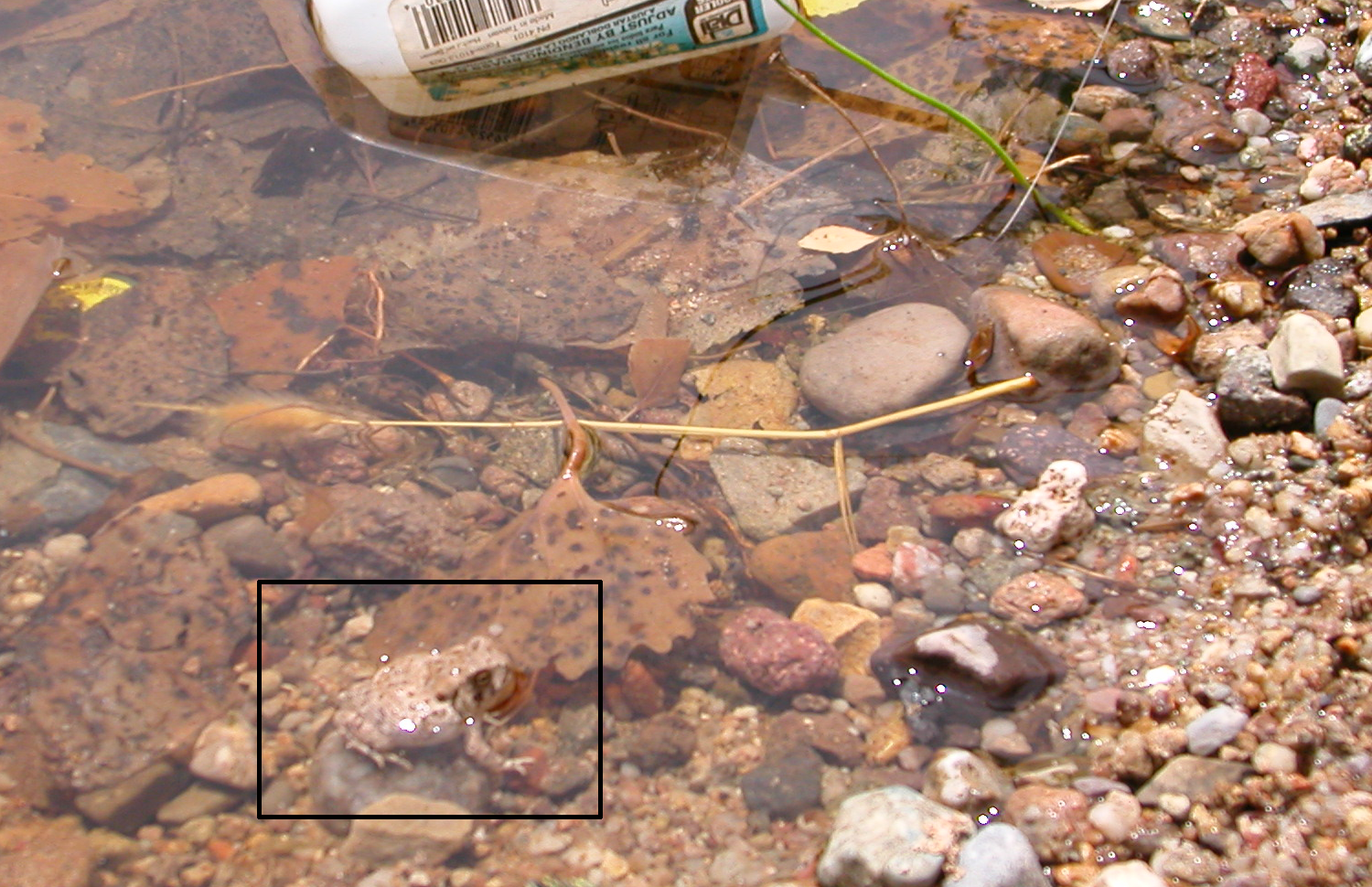

Supplement: Figure S9 — A toad using one of the artificial pools. (TIF) [file pone.0109276.s009.tif]
