# Supplementary material for: Sensitivity and Tolerance of Riparian Arthropod Communities to Altered Water Resources along a Drying River
Source: PLoS One. 2014 Oct 8;9(10):e109276. doi: 10.1371/journal.pone.0109276 (PMC4190312; doi:10.1371/journal.pone.0109276)
Supplement: Table S1 — Comparison of results with and without including 2 sites (one pool and one dry) with uncharacteristic distributions of carabid beetles and/or ants. (DOCX) [file pone.0109276.s010.docx]

Table S1. Comparison of results with and without including 2 sites (one pool and one dry) with uncharacteristic distributions of carabid beetles and/or ants.

|  | **Limited Dataset (In paper, n=6)** | | **Full Dataset (For comparison n= 7)** | |
| --- | --- | --- | --- | --- |
| **Response** | **Test Statistic** | **p-value** | **Test Statistic** | **p-value** |
| Total abundance | | | | |
| Change in Total Abundance | χ^2^ = 0.05 | P = 0.826 | χ^2^ = 0.31 | P = 0.578 |
| Trophic Groups | | | | |
| Change in Composition (Abundance) | F = 0.64 | P = 0.571 | F = 0.34 | P = 0.785 |
| Change in Composition (Biomass) | F = 1.67 | P = 0.070 | F = 1.46 | P = 0.149 |
| Change in Predator Abundance | χ^2^ = 0.02 | P = 0.889 | χ^2^ = 0.06 | P = 0.800 |
| Predator Biomass Over Time | χ^2^ = 10.07 | P = 0.002 | χ^2^ = 5.98 | P = 0.015 |
| Shannon’s Diversity Over Time | χ^2^ = 9.33 | P = 0.002 | χ^2^ = 5.41 | P = 0.020 |
| Pielou’s Evenness Over Time | χ^2^ = 6.72 | P = 0.010 | χ^2^ = 3.64 | P = 0.056 |
| Richness Over Time | χ^2^ = 7.77 | P = 0.005 | χ^2^ = 7.05 | P = 0.008 |
| Orders | | | | |
| Change in Composition (Abundance) | F = 1.55 | P = 0.197 | F = 0.93 | P = 0.465 |
| Change in Composition (Biomass) | F = 2.52 | P = 0.001 | F = 1.75 | P = 0.076 |
| Final Composition (Biomass) | F = 4.66 | P = 0.025 | F = 2.35 | P = 0.088 |
| Coleoptera Biomass Over Time* | χ^2^ = 8.25 | P = 0.004 | χ^2^ = 2.34 | P = 0.126 |
| Shannon’s Diversity Over Time* | χ^2^ = 5.00 | P = 0.025 | χ^2^ = 0.03 | P = 0.858 |
| Families | | | | |
| Change in Composition (Abundance) | F = 1.62 | P = 0.134 | F = 0.713 | P = 0.635 |
| Change in Composition (Biomass) | F = 2.64 | P = 0.002 | F = 1.80 | P = 0.071 |
| Final Composition (Biomass) | F = 3.40 | P = 0.030 | F = 1.89 | P = 0.114 |
| Carabidae Biomass Over Time* | χ^2^ = 7.68 | P = 0.006 | χ^2^ = 2.10 | P = 0.147 |
| Lycosidae Biomass Over Time | χ^2^ = 9.52 | P = 0.002 | χ^2^ = 6.51 | P = 0.011 |
| Change in Noctuidae Biomass | W = 31.5 | P = 0.026 | W = 40 | P = 0.034 |
| Final Noctuidae Biomass | W = 31.5 | P = 0.026 | W = 40 | P = 0.034 |
| Shannon’s Diversity Over Time | χ^2^ = 6.00 | P = 0.014 | χ^2^ = 4.98 | P = 0.026 |
| Pielou’s Evenness Over Time | χ^2^ = 7.56 | P = 0.006 | χ^2^ = 4.56 | P = 0.033 |
| Carabidae Genera | | | | |
| Change in Composition (Abundance) | F = 4.23 | P = 0.012 | F = 2.28 | P = 0.089 |
| Final Composition (Abundance) | F = 5.69 | P = 0.012 | F = 2.05 | P = 0.132 |
| Change in Composition (Biomass) | F = 3.01 | P = 0.003 | F = 2.08 | P = 0.070 |
| Final Composition (Biomass) | F = 7.81 | P = 0.005 | F = 2.42 | P = 0.117 |
| Change in *Brachinus* Abundance | χ^2^ = 13.41 | P = 0.000 | χ^2^ = 6.98 | P = 0.037 |
| Final *Brachinus* Abundance | χ^2^ = 23.66 | P = 0.000 | χ^2^ = 11.56 | P = 0.055 |
| Change in *Chlaenius* Abundance | χ^2^ = 1.97 | P = 0.010 | χ^2^ = 1.01 | P = 0.102 |
| Final *Chlaenius* Abundance | χ^2^ = 1.05 | P = 0.253 | χ^2^ = 0.20 | P = 0.604 |
| *Brachinus* Biomass Over Time* | χ^2^ = 6.33 | P = 0.012 | χ^2^ = 1.88 | P = 0.170 |
| Change in *Chlaenius* Biomass | W = 31.5 | P = 0.026 | W = 37 | P = 0.102 |
| Final *Chlaenius* Biomass | W = 25.5 | P = 0.182 | W = 29 | P = 0.551 |
| Carabidae Genera Richness over Time* | χ^2^ = 2.34 | P = 0.037 | χ^2^ = 2.08 | P = 0.149 |

*****Time x treatment interaction. All others are treatment effect.
