# Supplementary material for: Sensitivity and Tolerance of Riparian Arthropod Communities to Altered Water Resources along a Drying River
Source: PLoS One. 2014 Oct 8;9(10):e109276. doi: 10.1371/journal.pone.0109276 (PMC4190312; doi:10.1371/journal.pone.0109276)
Supplement: Table S2 — Results of tests comparing artificial pools to flowing reference sites. (DOCX) [file pone.0109276.s011.docx]

**Table S2. Results of tests comparing artificial pools to flowing reference sites.**

| **Response** | **Test Statistic** | **df** | **p-value** |
| --- | --- | --- | --- |
| Composition of aquatic orders | F = 7.57 | 1, 9 | 0.009 |
| Abundance of aquatic insects | χ^2^ = 4.26 | 1 | 0.039 |
| Composition of terrestrial orders | F = 2.38 | 1, 13 | 0.035 |
| Composition of terrestrial families | F = 1.37 | 1, 13 | 0.216 |
| Composition of carabid beetle genera | F = 20.41 | 1, 13 | 0.001 |
| Final total terrestrial abundance | χ^2^ = 14.13 | 2 | 0.001 |
| Final total terrestrial biomass | χ^2^ = 6.17 | 2 | 0.009 |
| Final bombardier beetle abundance | χ^2^ = 63.30 | 2 | <0.001 |
| Final bombardier beetle biomass | χ^2^ = 9.55 | 2 | 0.001 |
