# Supplementary material for: Sensitivity and Tolerance of Riparian Arthropod Communities to Altered Water Resources along a Drying River
Source: PLoS One. 2014 Oct 8;9(10):e109276. doi: 10.1371/journal.pone.0109276 (PMC4190312; doi:10.1371/journal.pone.0109276)
Supplement: Text S1 — Supplementary Methods. (DOC) [file pone.0109276.s012.doc]

**Text S1. Supplementary Methods**

When creating pools, we stratified treatment assignment rather than randomizing, in order to keep dry sites equidistant from pools and pools equidistant from each other and make the experiment feasible given labor constraints.

To fill and maintain the pools, 121 L (32-gal) Rubbermaid™ trashcans were connected, via polyvinyl tubing, to water heater style float valves mounted on stakes in the pools. These trashcans were placed several meters away on the highest nearby area to provide sufficient gravity-induced pressure for water to flow into the pools. Trashcans were topped with loose fitting lids to reduce evaporation and debris buildup (Fig 1). Throughout the experiment, pools were replenished by refilling trashcans with potable well water taken from Boquillas Ranch, a nearby BLM research station.

The number 4 plastic used in the trashcans is considered nontoxic, but to check for water toxicity, 3 cages of adult field crickets were provided with water taken from the tanks, water taken from the pools, and well water. Although this trial had true replication of one and no quantitative data was taken, no clear die-offs were observed and survival did not appear to differ after several days. Additionally, the presence of aquatic insects in all pools and the unexplained appearance and survival of mosquito fish (*Gambusia affinis*) in one pool indicated a lack of toxicity.

Existing methods of pitfall sampling found in the literature were not appropriate for this study. Sampling using soapy water may attract water stressed organisms and thus could potentially give artificially high numbers at dry sites as compared to pool and flowing sites (K. McCluney et al, unpublished data). Trapping with ethylene glycol can have unintended consequences for mammals that may drink from the traps (including death). Dry pitfall traps with refuges were used for the first sampling event. However, out of 88 traps, zero crickets were captured. Other studies showed that these low abundances were not appropriate (Sabo et al. 2005a) and thus it is likely that crickets were escaping from the traps or being eaten. Thus we used sticky pitfall traps for all remaining sampling events. Although we set two pitfall traps per site, one trap on one date was destroyed (Pool 7 on 25 June 2006). Because of that loss and a desire to simplify the statistical analyses, we examine mean values for each site, standardizing to per trap values.

After sampling, cups were returned to Ziploc bags and frozen. Frozen samples were later thawed and filled with baby oil to dissolve Tanglefoot™ and loosen samples. Traps were allowed to soak at ambient shaded field temperatures (in an enclosed shed) for 24-48 hours at which point, trapped organisms were poured back into the Ziploc bags. Any remaining organisms were removed from the cups with tweezers and added to the bags. Bags were stored for a short period in a freezer, then for several months in a refrigerator, and then for 2 more years in a freezer.

Our methods of pitfall trapping have drawbacks, including increased processing time and decreased sample quality, but are likely to be less biased than other methods, especially when trapping across a gradient of water availability (K. McCluney et al, unpublished data). All pitfall trapping provides an estimate of activity-abundance and thus inherently has some bias. Due to biases in our sampling methods and our inability to identify small arthropods, we excluded all individuals smaller than 1.5 mm and all collembolans from analyses.

We calculated adult biomass based on published literature values. When possible, we used order specific equations, or a single generalized terrestrial arthropod equation when order specific equations were not available.
